# Imaging Diagnosis of Mild Cognitive Impairment: A Bibliometric Analysis (1999–2024)

**DOI:** 10.1002/brb3.71144

**Published:** 2025-12-22

**Authors:** Jianan Xie, Shuya Li, Kuncheng Li, Kai Sun

**Affiliations:** ^1^ Institute of Medical Imaging Longgang District Maternity & Child Healthcare Hospital of Shenzhen City (Affiliated Shenzhen Women and Children's Hospital (Longgang) of Shantou University Medical College) Shantou University Medical College Shenzhen China; ^2^ Guangdong Key Laboratory For Biomedical Measurements and Ultrasound Imaging, National‐Regional Key Technology Engineering Laboratory For Medical Ultrasound, School of Biomedical Engineering Shenzhen University Shenzhen China; ^3^ Department of Experiment and Research South China Hospital, Medical School,Shenzhen University Shenzhen China; ^4^ PET Center Zhongshan Hospital of Dalian University Dalian China; ^5^ Longgang Institute of Medical Imaging, Shantou University Medical College & The Third People's Hospital of Longgang District, Shenzhen Beijing China; ^6^ Department of Radiology and Nuclear Medicine Xuanwu Hospital, Capital Medical University Shenzhen China; ^7^ Shenzhen Clinical Medical School, Guangzhou University of Chinese Medicine, Shenzhen, 518116 China

**Keywords:** artificial intelligence, bibliometrics, mild cognitive impairment, neuroimaging

## Abstract

**Purpose:**

Imaging diagnosis of mild cognitive impairment (MCI) has garnered increasing attention due to its critical role in the early detection of Alzheimer's disease (AD) and other dementias. This study presents a bibliometric analysis to elucidate global research trends, key contributors, thematic clusters, and emerging topics within the field of MCI imaging diagnosis.

**Method:**

English‐language publications related to MCI imaging were retrieved from the Web of Science Core Collection (WoSCC) from January 1999 to December 2024. Bibliometric analyses were performed using VOSviewer, CiteSpace, and R‐bibliometrix to evaluate co‐authorship networks, institutional collaborations, journal impact, keyword co‐occurrence, and burst trends.

**Finding:**

A total of 7,568 articles showed an average annual growth of 22.27%, with output surging after 2007 and peaking in 2024 (n = 762). The United States led in productivity and impact, ahead of China and Italy. Leading institutions were the University of California System, Vrije Universiteit Amsterdam, and the University of London, with key authors including Clifford R. Jack Jr., Ronald C. Petersen, and Philip Scheltens. Core journals were Neurology, Neuroimage, and Brain. Cluster analysis revealed four themes: functional and cognitive networks, biomarkers and pathology, structural imaging and computational diagnostics, and guidelines. Recent trendsc (AI) (e.g., machine learning, deep learning), while citation bursts indicate an evolution from early biomarker and imaging research toward current AI and multimodal imaging for improved diagnosis and risk prediction.

**Conclusion:**

This bibliometric analysis provides a comprehensive overview of the evolving research landscape in MCI imaging diagnosis. The integration of advanced computational methodologies, particularly AI‐powered tools, is driving precision diagnostics and personalized medicine. These advancements hold significant potential to improve early detection, stratify risk, and inform therapeutic interventions, ultimately contributing to better outcomes for individuals with MCI.

## Graphical abstract

This study presents a bibliometric analysis of global research trends in imaging diagnosis of mild cognitive impairment (1999–2024), highlighting key contributors, neuroimaging advancements, and the growing role of artificial intelligence in early detection and diagnosis of cognitive decline.

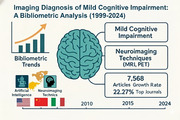

**Abbreviations**
AD: Alzheimer's disease
AI: artificial intelligence
Aβ‐PET: beta‐amyloid plaque molecular imaging
CNNs: convolutional neural networks
CT: computed tomography
EOAD: early‐onset Alzheimer's disease
fMRI: functional MRI
IF: impact factor
JCR: journal citation reports
LEADS: longitudinal early‐onset Alzheimer's disease study
MCI: mild cognitive impairment
MRI: magnetic resonance imaging
NMRI: nuclear magnetic resonance imaging
PET: positron emission tomography
SPECT: single‐photon emission computed tomography
TC: total citations
TP: total publications
WMHs: white matter hyperintensities
WoSCC: web of science core collection

## 1 Introduction

Mild cognitive impairment (MCI) is a clinical condition characterized by subtle cognitive deficits, particularly memory loss, in older adults, which do not yet interfere with daily functioning (Petersen et al. 2018). Primary symptoms include memory decline, reduced concentration, and changes in temperament, such as irritability, apathy, or mild depression. Although these symptoms are noticeable, they are insufficient for a diagnosis of dementia (Cheng et al. 2017). MCI is relatively prevalent among the elderly, especially those over 60, with prevalence rates ranging from 6.7% to 25.2% (Jongsiriyanyong and Limpawattana 2018). As populations continue to age, the incidence of MCI is expected to rise, posing a significant challenge to the health and quality of life of older adults (Petersen 2016). Furthermore, MCI not only affects individuals but also imposes a substantial burden on families and society, given that some MCI cases may progress to dementia over time. Neuroimaging, particularly functional imaging, can reveal brain structural and functional abnormalities that may be detectable before clinical cognitive deficits manifest (Yang et al. 2018).

The diagnosis of MCI requires a multifaceted approach to ensure accuracy, with neuroimaging playing a pivotal role (Anderson 2019). Techniques such as computed tomography (CT) and magnetic resonance imaging (MRI) allow clinicians to assess structural and functional brain changes, facilitating the identification of conditions like brain atrophy (Wang et al. 2023). Primary imaging modalities include CT, MRI, and positron emission tomography (PET) (Chandra et al. 2019). MRI is particularly favored in MCI research due to its non‐invasive nature, lack of ionizing radiation, and cost‐effectiveness. MRI demonstrates high diagnostic utility in distinguishing Alzheimer's disease (AD), MCI, and normal aging (Shahidi et al. 2023). It reveals atrophy and functional anomalies in brain regions such as the hippocampus and temporal lobes, with grey matter atrophy serving as an early marker of cognitive decline (Benedict et al. 2020). Advanced techniques, such as functional MRI (fMRI), are increasingly employed in MCI diagnosis (Zacková et al. 2021), while PET enables the identification of molecular biomarkers associated with MCI, including alterations in metabolism and blood flow (Noble and Scarmeas 2009). Comprehensive diagnosis integrates imaging findings with patient history, clinical symptoms, and cognitive assessments. Diagnostic criteria often include reported cognitive decline by patients or informants, clinician‐observed task difficulties or compensatory behaviors, and preserved daily functioning despite cognitive impairment (Mieling et al. 2023). In Western countries, imaging diagnostics are well‐developed, providing robust clinical evidence (Feng and Ding 2020; Veitch et al. 2024). Although imaging diagnostics in China began later, considerable progress has been achieved, with growing research efforts on MCI imaging (Wang et al. 2020; Zhong et al. 2023). However, imaging diagnostics have limitations, including high costs, technical complexity, and reliance on specialized personnel. Additionally, outcomes may vary depending on patient‐specific factors and disease progression. Therefore, a holistic approach integrating clinical symptoms and medical history is essential for enhancing diagnostic accuracy and reliability.

Bibliometric analysis, which employs mathematical and statistical methods to evaluate published research trends over time, provides a systematic overview of research categories, co‐authorship patterns, keyword frequencies, and the most‐cited articles or journals (Fresno‐Alba et al. 2022; Pei et al. 2022; Yuan et al. 2023). Currently, no bibliometric analysis has been conducted specifically on MCI diagnosis, underscoring the clinical relevance of this study. This approach aims to identify key research areas and emerging trends that could inform clinical practices and enhance diagnostic methods. Moreover, bibliometric analysis can highlight gaps in current diagnostic approaches, supporting the adoption and refinement of new technologies that may improve the early detection of cognitive impairments and mitigate disease progression. By providing a visual analysis of literature on MCI imaging diagnosis, this study aims to offer an intuitive understanding of the field's development, research hotspots, and cutting‐edge trends, contributing valuable insights to the advancement of neurology and gerontology.

## 2 Materials and Methods

### 2.1 Data Sources and Search Strategies

**FIGURE 1:**
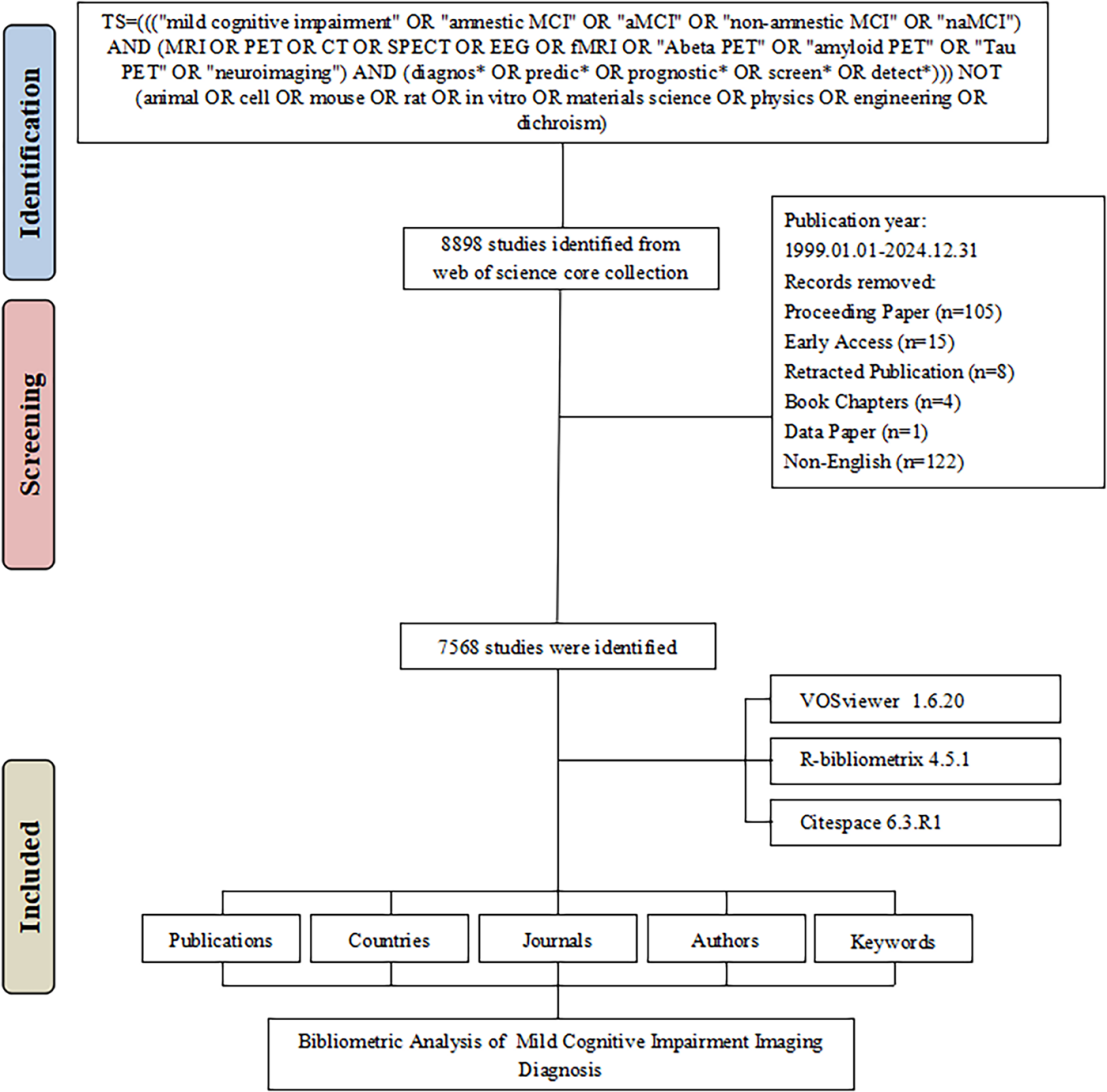
Flowchart of the literature screening process. This flowchart illustrates the step‐by‐step process of data screening and selection for bibliometric analysis in this study. Publications retrieved from the Web of Science Core Collection (WoSCC) were filtered using inclusion and exclusion criteria, resulting in a final dataset of 7,568 eligible articles. Key exclusion steps included removing non‐English publications, irrelevant studies, and specific publication types such as proceeding papers and retracted articles.

This bibliometric analysis utilized data from the Web of Science Core Collection (WoSCC, Clarivate Analytics), covering publications from January 1, 1999, to December 31, 2024. The WoSCC provides access to high‐impact journals across disciplines, forming a robust foundation for analyzing trends in imaging diagnosis for MCI. To ensure specificity and minimize noise, the search strategy included both inclusion and exclusion criteria. The inclusion criteria were (1) English‐language publications, (2) indexed in the SCIE or SSCI databases, and (3) publication type restricted to articles. The exclusion criteria were (1) non‐English publications, (2) proceeding papers, retracted publications, book chapters, data papers, and (3) studies unrelated to MCI imaging (e.g., animal or in vitro studies, materials science, or unrelated imaging modalities). The revised search formula was TS = (((“mild cognitive impairment” or “amnestic MCI” or “aMCI” or “non‐amnestic MCI” or “naMCI”) and (MRI or PET or CT or SPECT or EEG or fMRI or “Abeta PET” or “amyloid PET” or “Tau PET” or “neuroimaging”) and (diagnos* or predic* or prognostic* or screen* or detect*))) not (animal or cell or mouse or rat or in vitro or materials science or physics or engineering or dichroism). The final search was conducted on October 14, 2025, and retrieved 8898 publications. After applying the inclusion and exclusion criteria, 7568 eligible publications were retained for analysis, as illustrated in the updated data screening flowchart (Figure 1).

### 2.2 Data Collection and Visualization

The data extracted from the selected publications included titles, keywords, publication years, countries/regions, authors, institutions, journals, citation counts, and h‐index (Gao et al. 2017; Hirsch 2005; Synnestvedt et al. 2005). Data organization and basic statistical analyses were performed using Microsoft Excel. For visualizing collaboration networks, keyword co‐occurrence, and citation patterns, we employed three bibliometric tools: VOSviewer (version 1.6.20), CiteSpace (version 6.3.R1), and R‐bibliometrix (version 4.5.1). These tools provide complementary insights into research trends and collaboration dynamics within the field.

VOSviewer was used to construct co‐authorship and co‐citation networks, as well as to perform keyword co‐occurrence analyses. The parameters for each visualization are annotated in the respective figures to ensure methodological transparency. Specifically, the first two parameters in VOSviewer figures represent the number of included nodes and the minimum number of publications required for inclusion. The third and fourth parameters, “attraction” and “repulsion,” were adjusted to optimize the visualization layout, ensuring clarity and minimizing overlap between nodes. CiteSpace was employed for keyword burst analysis, with the following parameters: time slicing from 1999 to 2024 (1‐year intervals); node types set to “keywords”; and thresholds for the top 5 nodes per time slice. For pruning, the pathfinder and merged network pruning methods were applied to ensure clarity and relevance. R‐bibliometrix was used for an integrated analysis of bibliometric data, providing insights into publication trends, journal impact, and emerging research hotspots. The choice of parameter thresholds for CiteSpace and VOSviewer was guided by established practices in bibliometric research (Liu et al. 2022) and optimized to balance sensitivity and specificity. Although a formal sensitivity analysis was not performed, the stability of keyword clusters was ensured by iterative testing. Additionally, journal impact factors (IF) were reported based on JCR 2024 quartiles, reflecting the most up‐to‐date metrics at the time of analysis. Key bibliometric indices, including the h‐index and g‐index, were used to evaluate the productivity and citation impact of authors and journals. The h‐index reflects the number of articles (h) that have been cited at least h times, while the g‐index gives more weight to highly cited articles by ensuring the top g articles collectively receive at least g^2^ citations (Gao et al. 2017; Hirsch 2005; Synnestvedt et al. 2005).

## 3 Results

### 3.1 The Publication and Citation Trends

**FIGURE 2:**
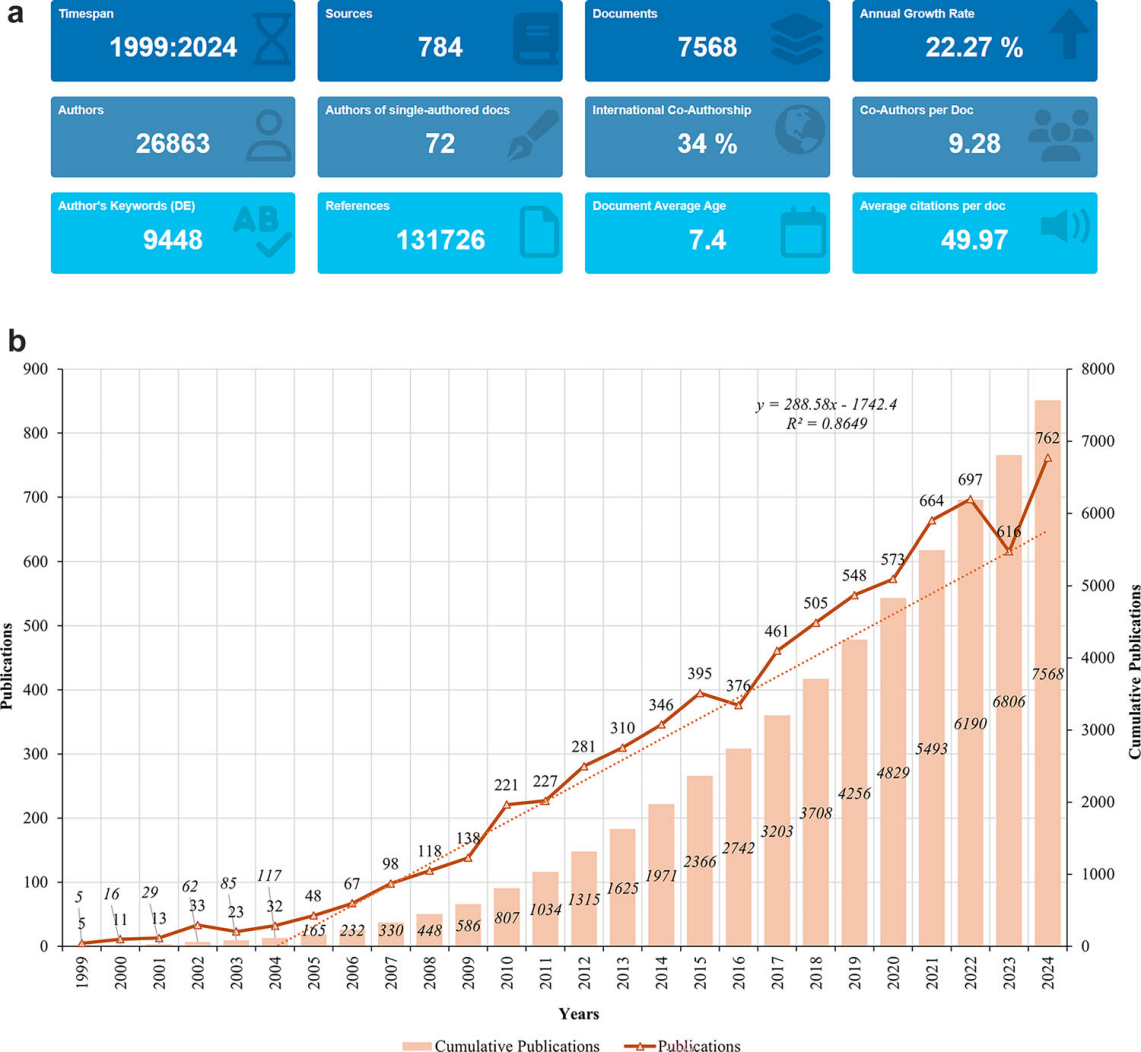
(a). Overview of publications, authors, institutions, and references. A schematic summary of the total number of publications, unique authors, institutions, journals, and references included in the bibliometric analysis of MCI imaging diagnostics. The figure highlights the global collaborative efforts and the extensive dataset analyzed from 1999 to 2024. (b). Annual publication trends. A line graph showing the annual number of articles published in the field of MCI imaging diagnostics. The graph demonstrates a steady increase in research output from 1999, with notable peaks in 2015 and 2024, reflecting the growing interest and advancements in the field.

In this bibliometric study, we conducted a comprehensive analysis of the scientific literature on MCI, with a focus on imaging diagnostics (Figure 1). A total of 7568 scholarly articles published between 1999 and 2024 were included after screening. These studies were authored by 26,863 researchers affiliated with 784 institutions across 104 countries and regions. Collectively, these articles were published in 784 sources and cited a total of 131,726 references, demonstrating the depth of collaboration and research in this field (Figure 2a). The dataset also includes a total of 9,448 distinct keywords, reflecting the diversity of topics explored. The annual growth rate of publications in this field was 22.27%, indicating a significant increase in research activity over time (Figure 2b). The number of annual publications remained relatively low before 2007, with fewer than 100 articles published yearly. However, a sharp increase was observed starting in 2008, with 165 articles, and this upward trend continued in subsequent years. Notable peaks include 548 publications in 2015, followed by a significant surge to a record high of 762 articles in 2024, marking a culmination of the field's growth. A slightly lower publication count of 616 articles in 2023 was followed by this unprecedented peak in 2024 with 7568 articles, reflecting the field's strong momentum. This growth trajectory, along with a high average citation rate of 49.97 citations per document, underscores the field's academic and clinical importance.

In the present study, we identified the most influential literature within the field by examining highly cited articles. Notably, the most influential is the article titled “Current concepts in mild cognitive impairment” published in *Arch Neurol* (Petersen et al. 2001), garnering an impressive 3,838 citations. Following this, the article “Lecanemab in Early Alzheimer's Disease”, from *N Engl J Med*, has also made a substantial contribution, amassing 3,073 citations (van Dyck et al. 2023; Jack et al. 2018). Additionally, “Neuropathological alterations in Alzheimer disease”, published in *Cold Spring Harb Perspect Med*, has been cited 2505 times (Serrano‐Pozo et al. 2011).

### 3.2 Analysis of Leading Countries and Institutions

**FIGURE 3:**
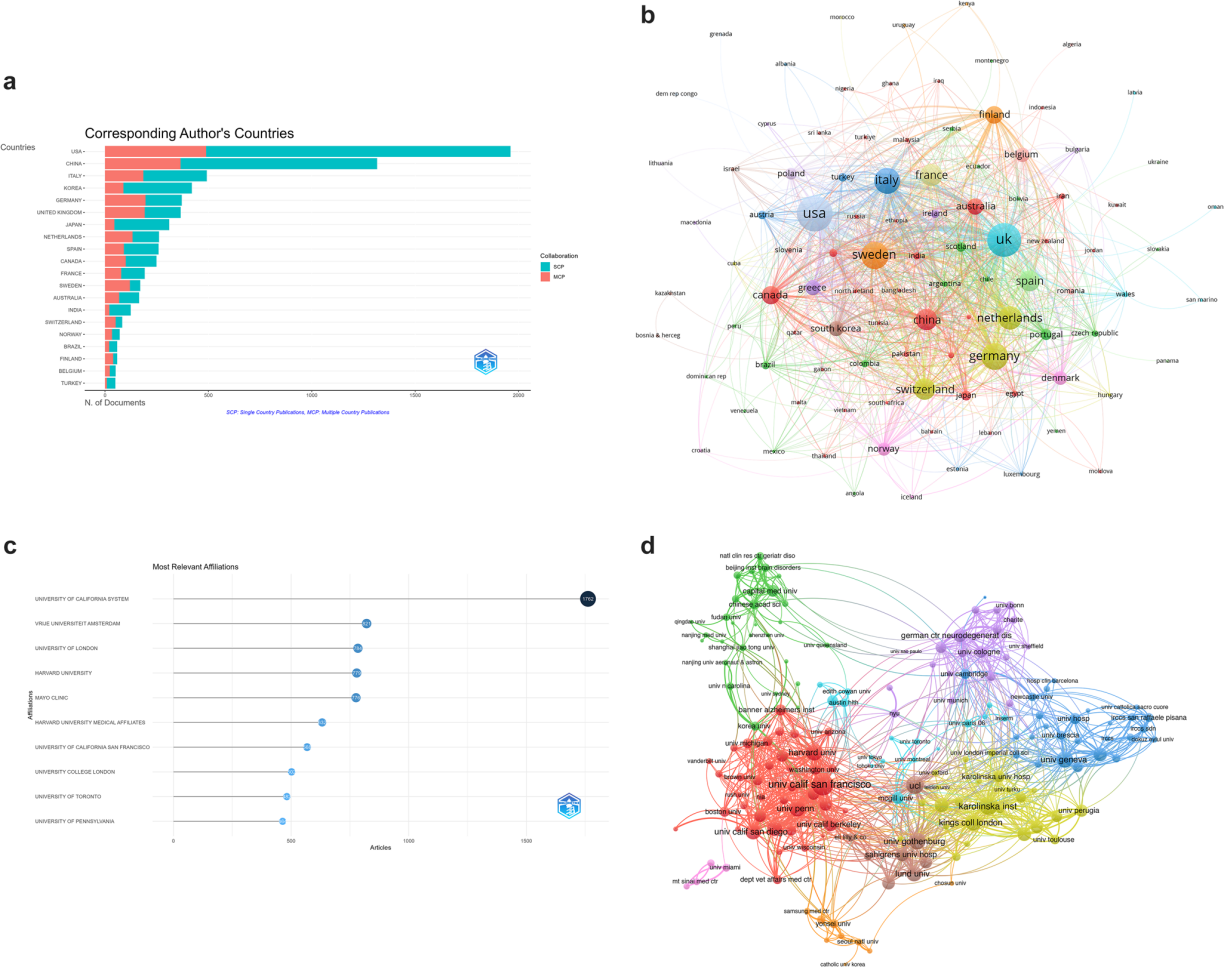
(a). Distribution of corresponding author's publications by country. A bar chart or world map showing the geographic distribution of publications based on the corresponding authors' affiliations. The USA, China, and Italy are identified as leading contributors to research in MCI imaging diagnostics. (b). Visualization map depicting the collaboration among different countries. A network visualization of international collaborations between countries in the field of MCI imaging diagnostics. Node sizes represent the number of articles produced by each country, while the thickness of connecting lines indicates the strength of collaborative ties. (c). Top ten institutions by article count and rank. A bar chart highlighting the ten institutions with the highest research output in MCI imaging diagnostics. The University of California System, Vrije Universiteit Amsterdam, and the University of London are shown as the most prolific institutions. (d). Visualization map depicting the collaboration among different institutions. A network visualization of institutional collaborations in the field of MCI imaging diagnostics. Node sizes represent institutional research output, and link thickness reflects the strength of collaboration. Institutions with at least 50 articles are included in the analysis.

The updated analysis of publication and citation profiles for leading countries in the field of MCI imaging diagnostics (Table 1 and Figure 3a) underscores substantial contributions from several nations. The USA remains the most prolific country, with 1,961 articles, accounting for 25.9% of the total output. It ranks first in both total publications (TP = 12,535) and total citations (TC = 158,671), with an impressive average of 80.9 citations per article, reflecting its strong academic influence and productivity. The USA also has the highest share of single‐country publications (SCP = 1472) and demonstrates notable international collaboration (MCP = 489), with a multiple‐country publication ratio (MCP Ratio) of 0.249. China follows as the second‐leading contributor, with 1,316 articles (17.4%), 5,583 total publications, and 32,876 citations, ranking second in both total publications and citations. However, its average citations per publication (25) remain significantly lower than that of the USA, indicating room for growth in academic influence. China's MCP ratio of 0.277 highlights its moderate engagement in international collaborations. Italy ranks third, with 492 articles (6.5%), 2,992 total publications, and 17,892 citations, securing fifth place in total citations. Italy's average citations per publication are 36.4, and it demonstrates a strong focus on international collaboration, as reflected by an MCP ratio of 0.376. In the realm of international research collaborations on MCI imaging diagnostics (Figure 3b), the USA has the highest total link strength (2398), followed by the UK (2150) and Sweden (1,372).

**TABLE 1:** Publication and citation profiles of leading countries.

Country	Articles	Freq	SCP	MCP	MCP_Ratio	TP	TP_rank	TC	TC_rank	Average Citations
USA	1961	0.259	1472	489	0.249	12535	1	158671	1	80.9
China	1316	0.174	951	365	0.277	5583	2	32876	2	25
Italy	492	0.065	307	185	0.376	2992	3	17892	5	36.4
Korea	420	0.055	331	89	0.212	2224	6	9779	10	23.3
Germany	371	0.049	175	196	0.528	2671	4	17326	6	46.7
UK	366	0.048	174	192	0.525	2273	5	19574	3	53.5
Japan	310	0.041	265	45	0.145	1413	11	8654	13	27.9
Netherlands	261	0.034	128	133	0.51	1585	8	17246	7	66.1
Spain	259	0.034	168	91	0.351	1451	10	8817	12	34
Canada	249	0.033	149	100	0.402	1655	7	9256	11	37.2
France	192	0.025	114	78	0.406	1513	9	13464	9	70.1
Sweden	170	0.022	50	120	0.706	1271	12	18794	4	110.6
Australia	165	0.022	97	68	0.412	1224	13	13912	8	84.3
India	124	0.016	103	21	0.169	303	18	2217	18	17.9
Switzerland	83	0.011	32	51	0.614	831	14	3245	15	39.1
Norway	71	0.009	37	34	0.479	485	15	2948	16	41.5
Brazil	59	0.008	39	20	0.339	274	20	1549	20	26.3
Finland	59	0.008	20	39	0.661	480	16	3716	14	63
Belgium	51	0.007	28	23	0.451	469	17	2304	17	45.2
Turkey	50	0.007	41	9	0.18	297	19	1273	21	25.5

*Note*(s): Articles: publications of corresponding authors only. Freq: frequence of total publications. MCP_Ratio: proportion of multiple country publications. TP: total publications. TP_rank: rank of total publications. TC: total citations. TC_rank: rank of total citations. Average Citations: the average number of citations per publication.

The analysis of the most relevant affiliations in the field of MCI imaging diagnostics, as depicted in Figure 3c, highlights the significant contributions from top institutions. The University of California System emerges as the leading institution, with a total of 1762 articles, underscoring its dominant role in advancing research output and innovation in this field. Following closely, Vrije Universiteit Amsterdam ranks second, contributing 821 articles, which reflects its prominent position in MCI imaging research. The University of London is also a major contributor, publishing 784 articles, further emphasizing its influence in the global research landscape. In the landscape of international research collaborations (Figure 3d), with a threshold of at least 50 articles, San Francisco takes the lead in collaborative efforts, engaging in 876 total link strength with institutions across the globe. The Karolinska institution comes next with 683 collaborations, and University College London, with a notable 627 collaborations, follows closely behind.

### 3.3 Analysis of Authors and Co‐Cited Authors

A total of 26,863 authors have contributed to this research field. The analysis of high‐impact authors in the field of MCI imaging diagnostics, as detailed in Table 2, highlights several leading researchers whose work has significantly influenced the scientific community. The top‐ranked author is Clifford R. Jack Jr., with an h‐index of 80 and a g‐index of 159. He has published 192 total papers since 1999, which have collectively garnered 25,779 total citations (TC), making him one of the most impactful contributors in this field. His m‐index of 2.963 reflects his sustained high‐quality research output over time. Ronald C. Petersen follows closely, with an h‐index of 76 and a g‐index of 140. He has published 140 papers, accumulating the highest total citation count (27,786). Petersen's m‐index of 2.815 further emphasizes the consistent influence of his work since 1999. In the third position is Philip Scheltens, who has an h‐index of 65 and a g‐index of 123. Scheltens has contributed 160 papers to the field, which have received 15,620 citations, reflecting his significant impact. His m‐index of 2.407 highlights his steady research contributions since 1999.

**TABLE 2:** Publication and citation profiles of high‐impact authors.

Author	h_index	g‐index	m‐index	PY_start	TP	TP_Frac	TP_rank	TC	TC_rank
JACK CR	80	159	2.963	1999	192	15.17	1	25779	2
PETERSEN RC	76	140	2.815	1999	140	13.53	4	27786	1
SCHELTENS P	65	123	2.407	1999	160	16.91	2	15620	5
WEINER MW	65	132	2.5	2000	140	11.92	4	17537	4
TROJANOWSKI JQ	56	79	2.667	2005	79	7.61	15	15065	6
BLENNOW K	55	105	3.056	2008	105	8.51	8	20012	3
KNOPMAN DS	55	93	2.5	2004	93	7.37	10	12023	11
BARKHOF F	53	89	2.409	2004	118	11.07	6	8290	15
SHEN DG	53	85	3.118	2009	85	18.27	13	9611	13
FRISONI GB	51	93	2.125	2002	142	11.2	3	9267	14
JAGUST WJ	51	68	1.962	2000	68	8.44	17	13780	8
SHAW LM	51	86	3	2009	86	6.24	12	13491	9
VAN DER FLIER WM	51	89	2.125	2002	106	11.34	7	8077	16
HANSSON O	49	81	2.882	2009	81	7.9	14	13406	10
ZETTERBERG H	49	89	2.882	2009	89	6.92	11	13935	7
THOMPSON PM	48	85	2.182	2004	99	10.4	9	7404	18
SOININEN H	46	77	2.091	2004	78	5.86	16	5959	20
BOEVE BF	42	61	1.556	1999	61	5.13	20	10783	12
JOHNSON KA	41	64	2.158	2007	64	4.62	18	6444	19
SPERLING RA	41	63	2.05	2006	63	5.33	19	7577	17

*Note*(s): H_index: The h‐index of the journal, which measures both the productivity and citation impact of the publications. g_index: The g‐index of the journal, which gives more weight to highly‐cited articles. m_index: The m‐index of the journal, which is the h‐index divided by the number of years since the first published paper.

Abbreviations: TP: total publications. TP_rank: rank of total publications. TC: total citations. TC_rank: rank of total citations. Average Citations: the average number of citations per publication. PY_start: publication year start, indicating the year the journal started publication.

**FIGURE 4:**
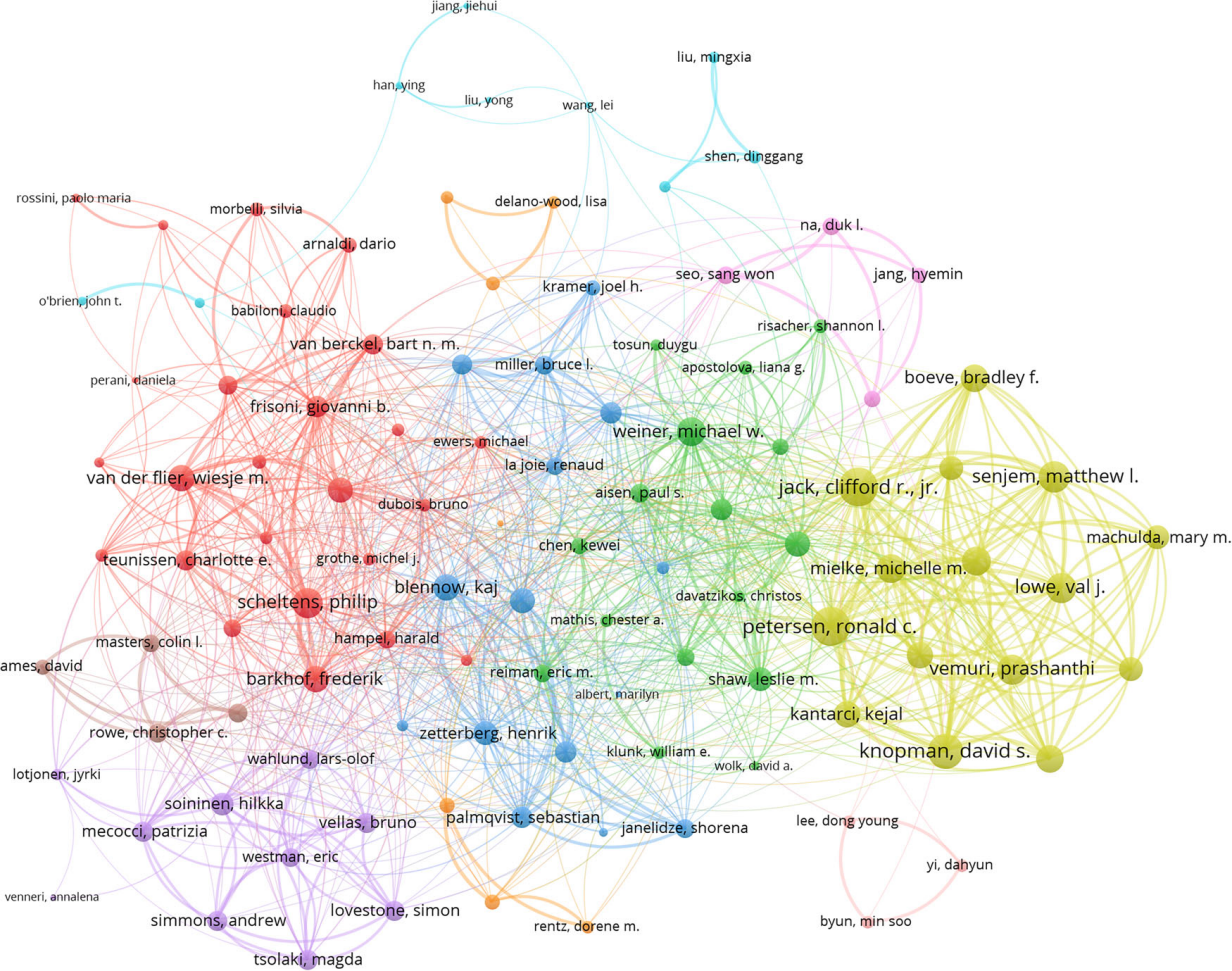
Visualization map depicting the collaboration among different authors. A network diagram showing co‐authorship patterns among the most prolific and influential authors in MCI imaging diagnostics. Node size indicates the number of publications by each author, and link thickness represents the intensity of collaboration. Authors with a minimum of 25 articles are included.

In the realm of international research collaborations within the field of MCI imaging diagnostics, a select group of 108 authors, each with a minimum of 25 articles, stands out for their cooperative efforts (Figure 4). Prominently, Clifford R Jr. Jack leads in the extent of his international collaborations, with a total link strength of 671, indicating a robust network of partnerships across different countries. This is followed by Ronald C. Petersen, whose collaborations have a total link strength of 664, and David S. Knopman, with a link strength of 532.

### 3.4 Analysis of Journals and Co‐Cited Journals

The bibliometric analysis of high‐impact journals in the field of MCI imaging diagnostics identifies several key publications that have significantly contributed to the advancement of the field (Table 3). *Neurology* leads with the highest h‐index of 100, reflecting its substantial scientific influence, and boasts an IF of 8.5, placing it in the prestigious Q1 category of the 2024 JCR Quartile. It has published 236 total articles, ranking fifth in terms of TPs, and has accumulated 25,674 TC, ranking second overall. *NeuroImage* follows closely, with an h‐index of 90 and an IF of 4.5, also in the Q1 category. The journal has published 240 articles (TP), ranking fourth in total publications, and leads the field with 27,663 citations (TC), making it the most influential journal in terms of citation impact. *Brain* ranks third with an h‐index of 76, an IF of 11.7, and a Q1 ranking. It has published 110 articles, ranking twelfth in total publications, but its 10,906 citations secure it fifth place in total citations, reflecting its strong academic impact despite a smaller number of publications.

**TABLE 3:** Bibliometric indicators of high‐impact journals.

Journal	H_index	G_index	M_index	IF 2024	JCR 2024	TP	TP_rank	TC	TC_rank	PY_start
Neurology	100	177	3.704	8.5	Q1	236	5	25674	2	1999
Neuroimage	90	159	3.6	4.5	Q1	240	4	27663	1	2001
Brain	76	110	3.167	11.7	Q1	110	12	10906	5	2002
Neurobiology of aging	72	121	2.769	3.5	Q2	263	3	12087	4	2000
Journal of alzheimers disease	65	96	2.708	3.1	Q2	678	1	9449	6	2002
Alzheimers & dementia	54	106	3	11.1	Q1	169	7	13871	3	2008
Neuroimage‐clinical	49	77	3.5	3.6	Q1	191	6	3062	20	2012
Plos one	48	85	2.526	2.6	Q2	161	9	5019	11	2007
Jama neurology	47	57	3.615	21.3	Q1	57	21	2403	27	2013
Human brain mapping	45	70	2.368	3.3	Q1	121	11	5771	10	2007
Journal of nuclear medicine	44	78	1.692	9.1	Q1	78	18	4105	15	2000
Frontiers in aging neuroscience	43	67	2.867	4.5	Q1	323	2	3109	18	2011
European journal of nuclear medicine and molecular imaging	40	68	1.739	7.6	Q1	106	13	2514	24	2003
Alzheimers research & therapy	38	54	2.533	7.6	Q1	165	8	2472	25	2011
Scientific reports	33	61	3	3.9	Q1	123	10	1896	35	2015
Dementia and geriatric cognitive disorders	32	48	1.185	1.9	Q3	93	15	3151	17	1999
Journal of neurology neurosurgery and psychiatry	32	41	1.28	7.5	Q1	41	32	4819	12	2001
Annals of neurology	30	42	1.25	7.7	Q1	42	29	6981	8	2002
Archives of neurology	30	33	1.2	NA	NA	33	41	8326	7	2001
Clinical neurophysiology	30	52	1.154	3.6	Q1	52	23	2829	22	2000

*Note*(s): H_index: The h‐index of the journal, which measures both the productivity and citation impact of the publications. IF: Impact Factor, indicating the average number of citations to recent articles published in the journal. JCR_Quartile: The quartile ranking of the journal in the Journal Citation Reports, indicating the journal's ranking relative to others in the same field (Q1: top 25%, Q2: 25%–50%, Q3: 50%–75%, Q4: bottom 25%).

Abbreviations: TP: total publications. TP_rank: rank of total publications. TC: total citations. TC_rank: rank of total citations. Average Citations: the average number of citations per publication. PY_start: publication year start, indicating the year the journal started publication.

**FIGURE 5:**
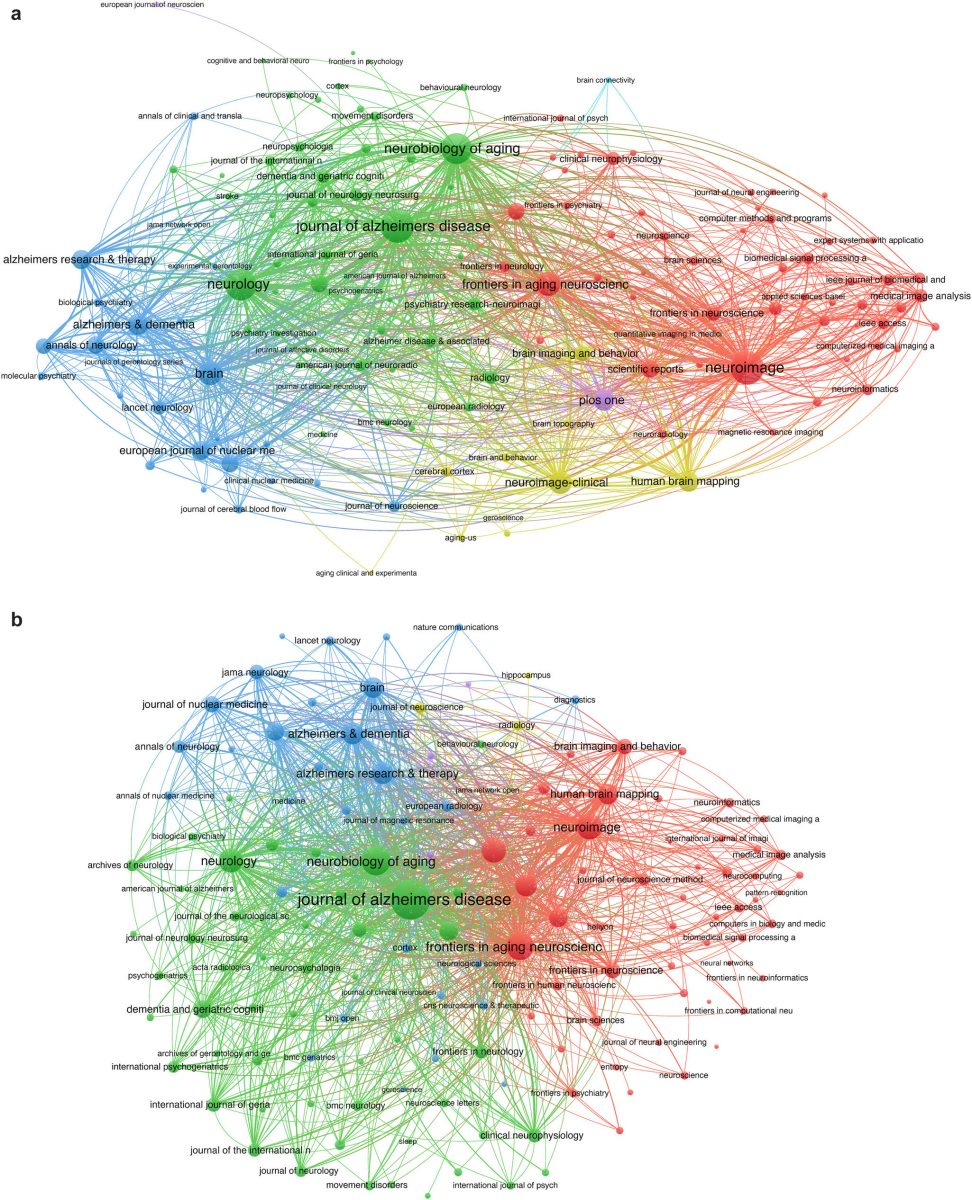
(a). Journal co‐occurrence network. A network visualization of journals contributing to the field of MCI imaging diagnostics. Node sizes indicate the number of articles published by each journal, and link strength reflects co‐occurrence or shared citations. Key journals include *NeuroImage*, *Neurology*, and *Brain*. (b). Coupling network of journals. A network map illustrating the interconnectedness of journals based on co‐citation analysis. Node size corresponds to citation volume, and link strength represents the extent of shared references between journals. The *Journal of Alzheimer's Disease* and *Neurobiology of Aging* are prominent contributors.

The analysis of co‐occurrence networks in the field of MCI imaging diagnostics reveals a robust framework of interconnected journals, with 133 journals having at least 15 occurrences (Figure 5a). The *Journal of Alzheimer's Disease* stands out with the highest total link strength, amassing 6838 connections. *Neuroimage* follows as a significant contributor, with a total link strength of 6397. *Neurology* also exerts considerable influence, evidenced by its total link strength of 5738. In the context of coupling networks, which measure the interconnectedness of journals based on co‐citation relationships, 140 journals are identified with a minimum of 10 connections (Figure 5b). The *Journal of Alzheimer's Disease* leads with an exceptional total link strength of 2,134,954, underscoring its pivotal position in the research landscape. *Neurobiology of Aging* and *Frontiers in Aging Neuroscience* also demonstrate their importance, with total link strengths of 1,050,705 and 925,376, respectively.

### 3.5 Analysis of Co‐Occurring Keywords and Burst Terms

**FIGURE 6:**
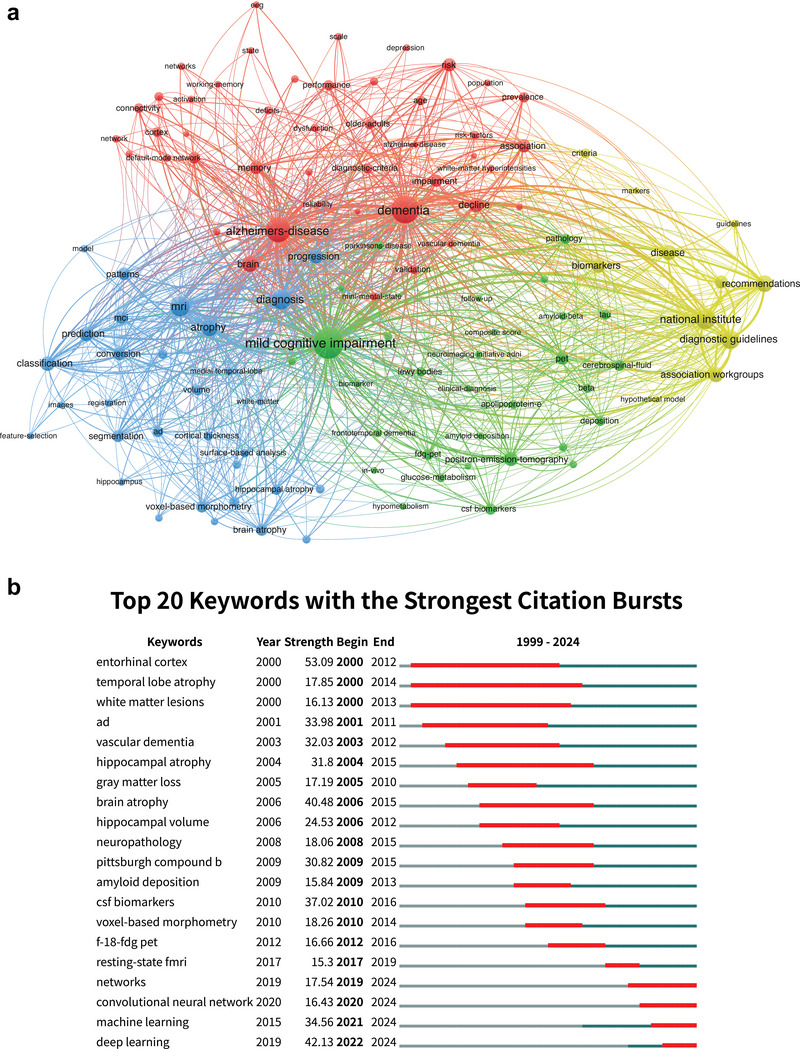
(a). Visual analysis of keyword co‐occurrence network analysis. A network visualization of co‐occurring keywords in the field of MCI imaging diagnostics. Keywords are grouped into thematic clusters, including functional and cognitive networks, biomarkers and pathological changes, structural imaging and computational diagnostics, and clinical guidelines and recommendations. Node size reflects keyword frequency, and link thickness indicates co‐occurrence strength. (b). Top 20 Keywords with the Strongest Citation Bursts. A timeline visualization showing the top 20 keywords with the strongest citation bursts over time. The figure highlights shifts in research focus, from early studies on biomarkers and structural imaging to recent trends emphasizing artificial intelligence and machine learning in MCI imaging diagnostics.

The co‐occurrence analysis of keywords in the field of MCI reveals a complex network of interconnected concepts that define the landscape of scientific inquiry (Table 4). The term “mild cognitive impairment” is the most prominent keyword, with 3,550 occurrences and a total link strength of 15,747, underscoring its foundational role in the field. Other significant keywords include “dementia” (2,609 occurrences, link strength 12,696) and “Alzheimer's disease” (2,379 occurrences, link strength 10,246), reflecting the close relationship between these conditions in the research landscape. The network visualization (Figure 6a) identifies four distinct clusters, each representing a thematic area of focus. These clusters, along with their summarized themes, are as follows: Cluster 1 (red): functional and cognitive networks. This cluster, comprising 45 items, focuses on functional and cognitive processes associated with MCI and AD. Key terms such as “connectivity,” “default‐mode network,” “working memory,” and “functional connectivity” highlight the emphasis on brain network dysfunction and cognitive decline. It also incorporates keywords like “age,” “depression,” and “risk factors,” which reflect the role of demographic and psychological variables in MCI progression. Cluster 2 (green): biomarkers and pathological changes. This cluster includes 31 items revolving around biomarkers and pathophysiological changes in MCI. Keywords like “amyloid‐beta,” “CSF biomarkers,” “tau,” and “glucose metabolism” underline the focus on structural and molecular markers. Additionally, terms such as “PET,” “cerebrospinal fluid,” and “frontotemporal dementia” emphasize imaging techniques and differential diagnosis. Cluster 3 (blue): structural imaging and diagnostic tools. This cluster, with 31 items, centers around structural imaging and diagnostic methodologies. Core keywords like “MRI,” “hippocampal atrophy,” “voxel‐based morphometry,” and “cortical thickness” reflect the use of advanced imaging techniques to study brain morphology. Terms such as “classification,” “segmentation,” and “prediction” indicate the integration of computational tools for automated diagnosis and risk assessment. Cluster 4 (yellow): guidelines and recommendations. This smaller cluster of 11 items focuses on clinical guidelines and diagnostic frameworks. Keywords such as “diagnostic guidelines,” “recommendations,” “criteria,” and “national institute” highlight the importance of standardization and consensus in MCI diagnosis.

**TABLE 4:** Top 20 keywords in mild cognitive impairment and Alzheimer's disease research by frequency and collaborative link strength.

Keyword	Occurrences	Total link strength
Mild cognitive impairment	4376	17492
Dementia	3214	14276
Alzheimers‐disease	2878	11249
National institute	1418	7824
Diagnosis	1702	6674
Diagnostic guidelines	1115	6238
Mri	1268	6011
Recommendations	808	4733
Atrophy	884	4290
Association workgroups	728	4085
Brain	932	3993
Disease	771	3587
Biomarkers	678	3237
Decline	651	3134
Progression	647	3124
Classification	711	2878
Association	615	2741
Risk	645	2636
Prediction	561	2617
Alzheimers association workgroups	440	2542

The analysis of keywords with the strongest citation bursts (Figure 6b) further demonstrates the complex interrelationships among these keywords. During the late 2000s and early 2010s, the field saw a surge in interest in structural and molecular imaging markers, with strong bursts for keywords such as “brain atrophy” (burst strength: 40.48, 2006–2015), “CSF biomarkers” (37.02, 2010–2016), and “Pittsburgh compound B” (30.82, 2009–2015). These keywords reflect a period of intensive research focused on validating diagnostic criteria and understanding pathological changes in MCI. In recent years, however, there has been a dramatic shift toward computational and artificial intelligence‐related topics. Keywords such as “deep learning” (burst strength: 42.13, 2022–2024), “machine learning” (burst strength: 34.56, 2021–2024), and “convolutional neural network” (burst strength: 16.43, 2020–2024) have emerged as dominant themes.

## 4 Discussion

### 4.1 General Information

This bibliometric study provides a thorough analysis of the scientific discourse on MCI, emphasizing imaging diagnostics. The examination underscores a robust collaborative framework, with a global network of authors and institutions contributing to a rich corpus of scholarly articles. The significant upward trend in the annual number of publications on MCI imaging diagnostics, with an annual growth rate of 29.05%, indicates expanding interest and advancements in this field. The peaks in publication counts, particularly in 2022 and 2024, may be attributed to rapid progress in technology, especially the integration of machine learning and deep learning techniques (Chaudhary et al. 2022; Illakiya and Karthik 2023). These technological advancements have significantly enhanced the diagnostic capabilities and research methodologies in MCI imaging diagnostics. The eminence of *Neuroimage*, *Neurology*, and *JAMA Neurology* within the MCI imaging diagnostics field is a testament to the interdisciplinary and profound research that defines this area of study (Barisano et al. 2022; Dean et al. 2014; Liew et al. 2023). These journals serve as pivotal platforms for disseminating knowledge, as evidenced by the extensive publication and citation of their articles. This broad academic engagement and the rigorous peer‐reviewed process they represent not only highlight the multifaceted nature of MCI research but also lay a robust groundwork for the evolution of diagnostic techniques and therapeutic strategies.

The analysis of publication and citation profiles across various countries and institutions reveals a dynamic and collaborative research landscape in the field of MCI imaging diagnostics. The USA, China, and Italy have emerged as leading contributors, reflecting a robust research infrastructure and a strong focus on advancing diagnostic methodologies. Institutionally, the University of California System, Harvard University, and the University of London stand out as major hubs of research activity, each contributing significantly to the collective knowledge base. The roles are further amplified by the collaborative research efforts, such as the meta‐analysis conducted by a network of 89 institutions, which underscores the critical role of cerebral amyloid‐β aggregation in the early stages of AD (Jansen et al. 2015). This comprehensive review, which synthesizes individual participant data from 55 studies, focuses on the prevalence of amyloid pathology as detected by positron emission tomography or cerebrospinal fluid, a key aspect of MCI imaging diagnostics. The analysis reveals a significant correlation between amyloid pathology and factors such as age, APOE genotype, and the severity of cognitive impairment (Jansen et al. 2015). The study's most striking insight is the identification of a 20‐ to 30‐year prodromal period between the initial detection of amyloid positivity and the onset of dementia, a finding that is pivotal for grasping the trajectory of AD and for the strategic planning of preventative interventions (Jansen et al. 2015). These collaborations are crucial for the exchange of ideas, resources, and expertise, which are essential for driving innovation and progress in the field. The integration of such collaborative findings into the broader research landscape, particularly in the context of MCI imaging diagnostics, exemplifies the power of global cooperation in advancing our understanding of neurological conditions and informing the development of effective therapeutic strategies.

The author analysis within the domain of MCI imaging diagnostics highlights the influential contributions of leading researchers, such as Clifford R Jr. Jack, whose work is exemplified by the study on “White matter hyperintensities are higher among early‐onset AD (EOAD) participants than their cognitively normal and early‐onset non‐AD peers” (*Alzheimer's Dementia*, 2023) (Eloyan et al. 2023). This research underscores the role of white matter hyperintensities (WMHs) in EOAD, revealing a significant association between WMH burden and cognitive impairment, as well as with amyloid and tau burden (Eloyan et al. 2023). Clifford's involvement in this study, which is part of the Longitudinal Early‐Onset Alzheimer's Disease Study (LEADS), showcases his commitment to advancing the understanding of EOAD and its pathological markers. In addition, the seminal work by Ronald C. Petersen, “Current concepts in mild cognitive impairment” (*Arch Neurology*, 2001), has been instrumental in shaping the field's understanding of MCI as a transitional state between normal aging and AD (Petersen et al. 2001). This article has been widely cited for its comprehensive summary of the diagnostic criteria, clinical outcomes, and the potential for therapeutic intervention in MCI. Petersen's early insights have been foundational in guiding subsequent research and clinical trials, which continue to explore the complexities of MCI and its progression to AD. Together, the contributions of these authors, as indicated by their high h‐ and g‐indices, reflect their substantial impact on the field. Their work has not only enriched the scientific discourse but also propelled the development of diagnostic tools and therapeutic strategies for MCI.

### 4.2 Keyword Analysis and Research Trends

The co‐occurrence of “dementia” and “Alzheimer's disease” in the scientific literature underscores the well‐established trajectory from mild cognitive impairment (MCI) to advanced cognitive decline. MCI is widely recognized as a prodromal stage of AD, characterized by cognitive decline that exceeds normal aging but does not yet meet dementia criteria (Jongsiriyanyong and Limpawattana 2018; Davis et al. 2018). This highlights the urgency of understanding MCI's progression to AD and developing interventions to delay or halt this transition (Jongsiriyanyong and Limpawattana 2018). Early diagnosis remains critical, as reflected in the prominence of keywords like “MRI,” “biomarkers,” and “diagnostic guidelines,” which emphasize the importance of neuroimaging and molecular markers in identifying MCI and predicting the risk of AD (Feng and Ding 2020; Davis et al. 2018). The multidisciplinary nature of MCI research, involving neurology, psychiatry, geriatrics, and cognitive science, highlights the complexity of the condition and the need for a holistic diagnostic and therapeutic approach (Jongsiriyanyong and Limpawattana 2018).

#### 4.2.1 Thematic Shifts in Research: Cluster Analysis

The cluster analysis reveals the main thematic areas shaping MCI research. Cluster 1 (functional and cognitive networks) emphasizes the role of brain networks, functional connectivity, and cognitive decline in understanding MCI's progression. This cluster highlights key terms like “default‐mode network,” “connectivity,” and “working memory,” reflecting the growing interest in how network dysfunction contributes to cognitive impairment. Research here has advanced through the integration of functional MRI (fMRI) and network‐based models, providing insights into how specific brain regions interact and degrade over time. Cluster 2 (biomarkers and pathological changes) focuses on molecular and imaging biomarkers, such as “amyloid‐beta,” “tau,” and “CSF biomarkers.” This reflects the biomarker‐driven approach to MCI diagnosis, which integrates cerebrospinal fluid assays and PET imaging to detect amyloid and tau pathology. The inclusion of terms like “glucose metabolism” and “FDG‐PET” highlights efforts to assess metabolic dysfunction as an early indicator of cognitive decline. These advancements align with the adoption of the A/T/N classification framework, which incorporates amyloid (A), tau (T), and neurodegeneration (N) markers into diagnostic algorithms (Jiao et al. 2023). Cluster 3 (structural imaging and computational diagnostics) highlights the increasing reliance on structural imaging and computational tools for MCI diagnosis. Keywords like “MRI,” “hippocampal atrophy,” “voxel‐based morphometry,” and “classification” reflect the use of high‐resolution MRI and volumetric analysis to detect neurodegenerative changes, particularly in the hippocampus and entorhinal cortex (Lombardi et al. 2020). Moreover, terms like “prediction,” “segmentation,” and “machine learning” signify the growing integration of artificial intelligence (AI) in analyzing imaging data, which has enhanced diagnostic precision and risk stratification (Basaia et al. 2019; Hu et al. 2021). Cluster 4 (guidelines and recommendations) focuses on standardizing diagnostic frameworks, as seen in keywords like “diagnostic guidelines,” “recommendations,” and “national institute.” This cluster reflects efforts to create consensus criteria for MCI diagnosis, particularly through contributions from organizations like the National Institute on Aging and Alzheimer's Association, which have advocated for biomarker‐driven approaches in clinical practice.

#### 4.2.2 Research Trends: Insights From Burst Keywords

The analysis of burst keywords provides a temporal lens to the evolving research priorities in MCI imaging diagnostics. During the early 2010s, a focus on structural and molecular markers is evident, with strong bursts for keywords like “Pittsburgh compound B” (reflecting amyloid PET imaging), “CSF biomarkers,” and “brain atrophy.” These trends underscore a period of intensive research aimed at identifying early pathological changes in AD and refining diagnostic accuracy (Jiao et al. 2023; Lombardi et al. 2020). The prominence of “neuropathology” and “glucose metabolism” during 2010–2016 reflects a deeper exploration of the mechanisms underlying MCI. Advances in FDG‐PET imaging and postmortem analyses enabled detailed assessments of cerebral metabolism and pathological hallmarks (Jalal et al. 2023; Pignalosa et al. 2021). These studies laid the foundation for multimodal imaging approaches, integrating structural, metabolic, and molecular data to refine disease models and patient stratification.

More recently, there has been a dramatic shift toward AI‐driven methodologies, as evidenced by the burst keywords “machine learning,” “deep learning,” and “convolutional neural network.” These trends align with advancements in computational power, algorithm development, and the availability of large‐scale neuroimaging datasets (Basaia et al. 2019; Hu et al. 2021; Feng et al. 2022). AI has significantly enhanced diagnostic capabilities by efficiently analyzing complex, high‐dimensional imaging data, improving diagnostic accuracy, predicting disease progression, and identifying novel imaging biomarkers. For example, convolutional neural networks (CNNs) have shown superior performance in classifying MCI and AD using MRI data (Basaia et al. 2019), while machine learning models have been applied to predict cognitive decline and stratify risk among elderly individuals (Hu et al. 2021). Deep learning approaches, such as multiview learning, have further identified imaging‐driven MCI subtypes that were previously undetectable using conventional methods (Feng et al. 2022; Wang et al. 2024).

Despite these advances, significant challenges remain in translating AI‐driven approaches into clinical practice. Key issues include the heterogeneity of imaging data across different sites, the limited generalizability of AI models trained on homogeneous cohorts, and the need for algorithms that are interpretable and trustworthy for clinicians. Addressing these challenges will require collaborative efforts to standardize imaging protocols, promote data sharing, and develop explainable AI methodologies. Nevertheless, the growing prominence of AI‐related keywords underscores the field's shift toward precision diagnostics and personalized medicine, leveraging computational innovation to address long‐standing clinical challenges. These trends highlight the integration of cutting‐edge technologies and biomarkers into MCI research, reflecting a more targeted and specialized approach to understanding and diagnosing cognitive impairments. Importantly, these advancements hold significant clinical implications, as they are poised to enhance earlier and more accurate detection of MCI, improve risk stratification, and ultimately inform the development of personalized therapeutic interventions.

### 4.3 Significance and Limitations

This bibliometric analysis provides valuable insights into the research landscape of imaging diagnostics for MCI, aiding researchers and clinicians in identifying key contributors, influential publications, and emerging trends. By mapping active research areas, the findings can support the adoption of new diagnostic methodologies and inform clinical practice to improve patient care. Moreover, the integration of AI and advanced imaging technologies reflects the growing emphasis on precision diagnostics, which has the potential to revolutionize how MCI is detected and managed.

However, several limitations of this study should be acknowledged. First, the analysis was restricted to articles indexed in the WoSCC, which may exclude relevant studies from other major databases, such as Scopus or PubMed. This reliance on a single database introduces the risk of database selection bias, potentially leading to an incomplete representation of the field. Second, the focus on English‐language publications may result in language bias, underrepresenting high‐quality research published in other languages. Additionally, journal selection bias may be present, as WoS primarily indexes journals with higher impact factors, potentially overlooking significant contributions from regional or emerging journals. The exclusion of non‐article publication types, such as book chapters and editorials, may omit important perspectives or novel hypotheses. Furthermore, the keyword‐based approach to bibliometric analysis may overlook emerging trends that use alternative terminology or synonyms. The lack of sensitivity analysis in keyword clustering is another limitation, although iterative testing was performed to ensure stability. To address these limitations in future studies, integrating data from multiple databases and including broader language and publication‐type criteria would provide a more comprehensive analysis. Additionally, conducting sensitivity analyses and incorporating emerging terms into keyword searches would enhance the robustness and depth of bibliometric findings.

## 5 Conclusion

In conclusion, this bibliometric analysis has thoroughly examined the field of imaging diagnosis for MCI, revealing a robust and evolving research landscape. The study has identified key trends, leading contributors, and influential journals that have shaped the discourse on MCI. The focus on neuroimaging and cognitive assessment, coupled with the integration of machine learning and deep learning techniques, marks a significant progression in the field. To further advance research and clinical practice, we recommend that future efforts prioritize fostering international collaborations, which can facilitate data sharing, increase sample diversity, and enhance scientific rigor. Additionally, the standardization of imaging protocols and reporting guidelines is essential for improving reproducibility, enabling meaningful multi‐center studies, and accelerating the translation of research findings into clinical practice. Funding agencies should consider supporting interdisciplinary projects that bridge neurology, computer science, and data engineering to address current challenges in artificial intelligence applications and model generalizability. The findings of this study offer a roadmap for future research, emphasizing the importance of interdisciplinary collaboration and the adoption of innovative methodologies. The integration of advanced computational methods into MCI imaging diagnostics is poised to enhance clinical practice and accelerate the discovery of novel therapeutic interventions. This study's contributions to the understanding of cognitive impairment are expected to assist in the refinement of diagnostic approaches and the improvement of treatment outcomes for individuals affected by MCI.

## Author Contributions

**Kai Sun**: conceptualization and design. **Kuncheng Li**: project administration. **Jianan Xie and Shuya Li**: data curation, formal analysis, investigation, writing – original draft preparation. All authors read and approved the final manuscript.

## Funding

The study was supported by Key Project of Medical and health technology of Longgang District, Shenzhen (grant number LGKCYLWS2022034). The funders had no role in study design, data collection, and analysis, decision to publish, or preparation of the manuscript.

## Ethics Statement

The authors have nothing to report.

## Data Availability

All data generated or analyzed during this study are included in this published article.
